# Estrogen, Cognitive Performance, and Functional Imaging Studies: What Are We Missing About Neuroprotection?

**DOI:** 10.3389/fncel.2022.866122

**Published:** 2022-05-12

**Authors:** Ivanny Carolina Marchant, Stéren Chabert, Jonathan Martínez-Pinto, Ramón Sotomayor-Zárate, Ricardo Ramírez-Barrantes, Lilian Acevedo, Claudio Córdova, Pablo Olivero

**Affiliations:** ^1^Laboratorio de Modelamiento en Medicina, Escuela de Medicina, Universidad de Valparaíso, Viña del Mar, Chile; ^2^Centro Interoperativo en Ciencias Odontológicas y Médicas, Universidad de Valparaíso, Valparaíso, Chile; ^3^Millennium Nucleus in Cardiovascular Magnetic Resonance, Santiago, Chile; ^4^Escuela de Ingeniería Biomédica, Universidad de Valparaiso, Valparaíso, Chile; ^5^Centro de Investigación y Desarrollo en Ingeniería en Salud, Universidad de Valparaíso, Valparaíso, Chile; ^6^Centro de Neurobiología y Fisiopatología Integrativa, Valparaíso, Chile; ^7^Laboratorio de Neuroquímica y Neurofarmacología, Facultad de Ciencias, Universidad de Valparaíso, Valparaiso, Chile; ^8^Instituto de Fisiología, Facultad de Ciencias, Universidad de Valparaíso, Valparaíso, Chile; ^9^Escuela de Tecnología Médica, Universidad Andres Bello, Viña del Mar, Chile; ^10^Servicio de Neurología Hospital Carlos van Buren, Valparaíso, Chile; ^11^Laboratorio de Estructura y Función Celular, Escuela de Medicina, Universidad de Valparaíso, Valparaíso, Chile

**Keywords:** estrogen (17β-estradiol), rapid effects of steroids, cognitive task, magnetic resonance imaging (MRI), cognitive performance, resting state—fMRI, neuroprotection

## Abstract

Menopause transition can be interpreted as a vulnerable state characterized by estrogen deficiency with detrimental systemic effects as the low-grade chronic inflammation that appears with aging and partly explains age-related disorders as cancer, diabetes mellitus and increased risk of cognitive impairment. Over the course of a lifetime, estrogen produces several beneficial effects in healthy neurological tissues as well as cardioprotective effects, and anti-inflammatory effects. However, clinical evidence on the efficacy of hormone treatment in menopausal women has failed to confirm the benefit reported in observational studies. Unambiguously, enhanced verbal memory is the most robust finding from longitudinal and cross-sectional studies, what merits consideration for future studies aiming to determine estrogen neuroprotective efficacy. Estrogen related brain activity and functional connectivity remain, however, unexplored. In this context, the resting state paradigm may provide valuable information about reproductive aging and hormonal treatment effects, and their relationship with brain imaging of functional connectivity may be key to understand and anticipate estrogen cognitive protective effects. To go in-depth into the molecular and cellular mechanisms underlying rapid-to-long lasting protective effects of estrogen, we will provide a comprehensive review of cognitive tasks used in animal studies to evaluate the effect of hormone treatment on cognitive performance and discuss about the tasks best suited to the demonstration of clinically significant differences in cognitive performance to be applied in human studies. Eventually, we will focus on studies evaluating the DMN activity and responsiveness to pharmacological stimulation in humans.

## Introduction

Menopause is a biological milestone linked to the onset of cognitive impairment, amongst several deleterious systemic effects. There would be a window of opportunity to provide hormone therapy (HT) that would benefit users. Clinical studies show cognitive improvements mainly in verbal memory (Maki, 2000) in women exposed to HT during this period. Studies are, however, hardly comparable due to heterogeneity in menopause origin, HT formulations, neuropsychological tests, and neuroimaging techniques. Experimental studies present differences in methodologies, type, and age of animal injury model that explain the apparent failure of scientific research to show the clinical benefit of estrogens.

Clinical studies have revealed higher activation of fronto-cingulate regions in menopausal women under estrogen treatment by brain functional magnetic resonance imaging (fMRI), although in many cases, no difference in cognitive performance was demonstrated. fMRI studies may also have different appreciations, thus the abnormal activation observed in some individuals who executed adequately the imposed task could be interpreted as a compensatory activation due to sub-clinical cognitive impairment (Comasco and Frokjaer, 2014).

During rest, synchronous hemodynamic activity occurs in different brain networks, the so-called Resting State Networks (RSN) (Ramírez-Barrantes et al., 2019), one of which being the default mode network (DMN). The DMN has been consistently implied as a biomarker of cognitive function and aging-related decline. Estrogen has been implicated in the modulation of DMN in pre and post-menopausal women (Petersen et al., 2014; Weis and Hodgetts, 2019). Resting state fMRI may thus be valuable to explore rapid estrogen effects avoiding confusion related to the application of cognitive tasks. According to the “healthy cell bias of estrogen benefit,” rapid estrogen effects in women with preserved DMN function may provide more insights into the complex nature of responsiveness to estrogen treatment cell bias of estrogen benefit (Brinton, 2008).

## From Animal Studies to the Understanding of Human Cognitive Functions

Sex hormones have important effects on non-reproductive organs such as the brain. Estrogen associates with the modulation of brain circuits involved in motivated behaviors, emotions, memory, and executive functions (Beyer, 1999; McEwen and Alves, 1999; Bethea et al., 2002; Almey and Milner, 2015). The relationship of plasma estrogen with these behaviors has been modeled in menopausal animals through ovariectomization (OVX) or by the administration of estrogen synthesis inhibitors. Several cognitive tasks modulated by estrogens have been extrapolated to human studies, either in young or post-menopausal women. We will expose examples of cognitive alterations observed in animals and humans and their relationship with estrogen therapy.

## OVX-Induced Cognitive Alterations

Ovariectomization is a safe surgery to produce undetectable serum levels of 17β-estradiol (E_2_) within 2–4 weeks (Medina-Contreras et al., 2020). Recently, in 5.5-month-old OVX rats, physical and psychological stressors were synergistic to decrease exploration, learning, and memory behaviors (Medina-Contreras et al., 2020). As E_2_ therapy reverses these alterations, a neuroprotective role of E_2_ has been suggested in post-menopausal women exposed to chronic stress (Khaleghi et al., 2021).

17β-estradiol effects are less intense in OVX administered in older (12 months) than middle-aged rats, since E_2_ only reversed anxious behaviors in the open field test without significant changes in the anxiety score in elevated plus maze or memory in novel object recognition test (Renczes et al., 2020). Chronic stress, chronic exposure to obesogenic, and hypercaloric diets can potentiate cognitive and behavioral alterations in OVX rats. In OVX macaques previously fed with a 6-week western diet, the animals treated with E_2_ for 30 months performed better spatial tasks than animals that received treatment via vehicle (Zimmerman et al., 2020).

## Drug Induced Menopause Produces Cognitive Alterations

4-vinylcyclohexene diepoxide (VCD) produces a gradual loss of ovarian follicles and E_2_ synthesis (Mayer et al., 2004). The 30-day VCD administration produces neurochemical alterations in monoamine and metabolite contents in the hippocampus, prefrontal cortex, and striatum, all of which express estrogen receptors (Long et al., 2019). E_2_ was more effective in restoring normal monoamine levels in the surgical model of menopause than in the VCD model (Long et al., 2019) and thus, it would be more effective in restoring cognitive performance in surgical menopause. The intracerebroventricular (icv) administration of the aromatase inhibitor letrozole produces dose-dependent cognitive alterations associated with a reduction in hippocampal E_2_ and a decrease in the firing rate of pyramidal neurons (Marbouti et al., 2020). Aromatase inhibition increases the expression of estrogen receptor α (Erα) and estrogen receptor β (Erβ) and decreases the expression of G protein-coupled estrogen receptor 1 (GPER) in the hippocampus (Marbouti et al., 2020), probably hampering memory formation. In humans, higher estradiol levels have ben correlated with greater hippocampal volume in men. Hippocampal activity has been reduced by letrozole, while a partially compensating increased prefrontal activity as in AD and aging that might mask estradiol's effect on observable behavior, i.e., memory performance, in some of the studies, can be observed. Subtle memory deficits in women under letrozole for therapeutical reasons may illuminate further analyses on these such E_2_-abstinence effects.

## Pathological Models Induced Cognitive Alterations

In 3-month-old rats, icv streptozotocin (STZ) produces similar memory and learning alterations to those observed in animal models of Alzheimer's disease (AD) (Wei et al., 2019). STZ induces oxidative alterations in the prefrontal cortex and hippocampus (Wei et al., 2019). The STZ administration, followed by chronic 21-day treatment with GPER agonist daidzein, increases the navigation time in the target quadrant and decreases the latency time to find the platform in Morris water maze (Wei et al., 2019). This study underlines the therapeutic potential of phytoestrogens in cognitive disorders (Echeverria et al., 2021).

The poly-I:C injection, a Toll-like receptor 3 agonist in pregnant mice, produces cognitive alterations in adult female offspring, which are reversed by the selective E_2_ receptor modulator raloxifene (Schroeder et al., 2019). Neonatal hypoxia due to carotid artery occlusion for 1 h at post-natal day 7 induces long-term cognitive deficits in rats (Anastacio et al., 2019) that are counteracted by phytoestrogen coumestrol until 3 h of hypoxia, as indicated by cognitive and morphological changes in hippocampus at post-natal day 60 (Hampson, 2018).

## Estrogens and Cognitive Tasks in Humans

The neuroprotective efficacy of HT remains unclarified. Considering that in the next decade, there would be 1.2 billion of menopausal and post-menopausal women (Hampson, 2018; Echeverria et al., 2021), it is necessary to standardize the cognitive task to be carried out in this population along with the pharmacological treatments to use. In humans, cognitive ability can be assessed by the mental rotation task (MRT) which considers perception, identification, orientation, judgment, response, and execution processes (Xue et al., 2017). In normal menstrual cycling women, luteal phase high progesterone levels are associated with better performance in visuospatial tasks in the MRT (Shirazi et al., 2021). In post-menopausal women with schizophrenia cognitive decline, the raloxifene administration did not produce significant changes in cognitive function compared to the group of patients receiving treatment via vehicle (Huerta-Ramos et al., 2020). Rather than associated with a loss of pharmacological HT effect, the diversity of human E_2_ effects should be correlated with psychiatric or neurodegenerative co-morbid conditions where the degree of cognitive deficit is variable in intensity and not necessarily reversed by neuromodulators.

## Receptor-Dependent Rapid Estrogen Mechanisms

The ability of estradiol to influence cognition during development, at adulthood, and during aging has been demonstrated years ago (Luine, 2012). E_2_ enhances *in vitro* the consolidation of hippocampal memories 5–30 min after treatment through the activation of several cell signaling cascades (Frick, 2015). E_2_ regulates the hippocampal morphology and function, spine density, neurogenesis, synaptic plasticity, neurotransmission, and gene expression, which are all facilitators of memory consolidation (Patel et al., 2022). It regulates the dendritic spine density in the medial prefrontal cortex, somatosensory cortex, amygdala, and dendritic length in the basal forebrain (Frick, 2015). E_2_ improves hippocampal-dependent spatial memory and object and spatial recognition memory in OVX rats.

As endocrine contributor, brain synthesizes E_2_ from androgen precursors by the enzyme aromatase, namely, the so-called neuron derived E_2_ (NDE_2_), found at synapses and presynaptic terminals in neurons in both male and female brains of rodents, monkeys, birds, amphibians, and humans. NDE_2_ regulates sexual differentiation, reproduction, synaptic plasticity, cognition, neuroinflammation, and neuroprotection (Brann et al., 2021). In the rat hippocampus, changing E_2_ levels across estrous phases suggest that CNS-synthesized E_2_ may be affected by the estrous cycle in rodents. Besides fluctuating hematic progesterone into the brain and its subsequent conversion into E_2_ (Kato et al., 2013), neural E_2_ changes might be related to changes of P450 aromatase depending on fluctuation of kinases related to synaptic plasticity (Hojo, 2018; Tozzi and Bellingacci, 2020).

In rat hippocampal slices treated with aromatase inhibitors, the amplitude of long-term potentiation is reduced with consequently reduced spatial, recognition, and contextual-fear memory, suggesting an important role of NDE_2_. NDE_2_ relates to synaptic plasticity and memory via the regulation of actin cytoskeleton polymerization/depolymerization and post-synaptic density dynamics, which is key for spine formation, enhancement of MAPK/ERK and PI3K-AKT signaling, regulation of CREB-BDNF signaling, and mediation by estrogen receptors and SRC-1 (Nelson, 2001; Spencer et al., 2008; Simpkins et al., 2012; Terasawa, 2018).

## Cognitive Tasks Assessments

Rapid E_2_ mechanisms on hippocampal memory in rodents have been most assessed in spatial tasks, i.e., Morris water maze, radial arm maze, delayed non-match to position, object placement task, and object recognition tasks. Other tools are social learning paradigms like the social transmission of food preferences, female mate choice copying, and social recognition (Zhao et al., 2017; Patel et al., 2022). ERα and ERβ agonists are also good alternatives to depict the molecular rapid E_2_ mechanisms. To observe rapid actions, E_2_ or ER agonists must be administered either systemically or intracranially minutes before the cognitive task since the rapid E_2_ effect expresses on neural plasticity within 1 h, modulating cell signaling, synaptic transmission, and dendritic spine density (Phan et al., 2011). ERα agonist propyl pyrazole triol (PPT) and ERβ agonist diarylpropionitrile (DPN) *in vitro* have shown to alter cell signaling, synaptic transmission, and long-term depression in hippocampal sections within 1.5 h of application (Phan et al., 2011). E_2_ can alter the performance in learning tests within 40 min of administration in OVX mice. Similarly, PPT administered to mice at 50 or 75 μg/mice s.c. 15 min before a social discrimination paradigm increased social recognition induced by ERα agonism. In addition, PPT or E_2_ improved object recognition and object placement learning (Phan et al., 2011, 2012; Gabor et al., 2015). Administered post training, systemically or into the dorsal hippocampus, E_2_ enhances spatial and object recognition memory consolidation in Morris water maze, spatial memory consolidation in an object location task, and object recognition memory consolidation (Gresack, 2006; Boulware and Heisler, 2013; Patel et al., 2022).

## Default Mode Network Activity and Functional Magnetic Resonance Imaging

Magnetic resonance images have been useful to explore brain functions for quite some time, as Ogawa et al. (1992) emphasized that the magnetic resonance (MR) signal, called “BOLD signal,” is blood oxygenation level dependent as observed in a task-related imaging set-up. The basis of BOLD signal is the ratio of oxy- vs. deoxy-hemoglobin in local venous blood that results from the rise in oxygen consumption and local blood flow when local neuronal activity increases. As the oxy- vs. deoxy-hemoglobin do not have the same paramagnetic properties, the MR signal is modified through a variation in ${\text{T}}_{2}^{*}$. It is noteworthy that the peak of signal variation is observed close to 5 s after the stimulus onset (Presa et al., 2020).

These first observations in fMRI are associated to the realization of a specific task of heterogeneous design that relies on patient cooperation with significant variability. A solution, as follows, was proposed by Biswal et al. (1995): with no special instruction to the patient but to just stay still, the time course of low-frequency fluctuation of some regions in the brain were shown to present high temporal correlation. Functional networks of different cortical regions were observed, identifying functionally connected nodes, such as the right and left motor cortex together, or the right and left visual cortex. As no stimulus was presented to the patient, the maps created were denominated resting-state functional MRI (rs-fMRI) (Damoiseaux et al., 2006). In either task-based fMRI or rs-fMRI, the precise physiological mechanisms underlying the temporal synchronous signal fluctuation between cortical areas are not yet clearly defined. The coupling or uncoupling of neuronal activity and vascular reactivity is an active field of study (Rossetti et al., 2021; Stiernman et al., 2021). The RSN is elicited by a wide variety of sensory, motor, and cognitive tasks, representing 20% of the overall energy consumed by a person (Raichle, 2010). It is composed by the salience network, executive network, auditory network, sensory motor network, visuospatial network, and default mode network (Smitha et al., 2017). The DMN is observed in awake individuals, containing areas mainly in the medial prefrontal cortex, and medial temporal lobe, and in the posterior cingulate cortex and angular gyrus (Buckner and Andrews-Hanna, 2008). Observations with other imaging modality, such as glucose imaging using positron emission tomography, supported the consideration that the DMN consists of specific areas connected in a stable network (Buckner and Andrews-Hanna, 2008). Exploration of DMN and its disruption has gained increasing interest in neuropsychiatric disorders as attention deficit hyperactivity disorder or ADHD (Mohan and Roberto, 2016). rs-fMRI provides identification of nodes that are functionally related and allows quantifying correlation, graph analysis, and functional connectivity analysis (Yang and Gohel, 2020). Further cognitive exploration can then be undertaken using different dynamic causal modeling (Friston, 2009). Various processing methods have been proposed in rs-fMRI with still no convergence on a standard one (Yang and Gohel, 2020). Seed-based connectivity analysis looks for correlation between regions-of-interest (ROI) with the associated question on how to define those ROI. Independent component analysis (ICA) can be applied in different manners, with the associated question on how many components must be considered and how to interpret each obtained component. There is also processing using amplitude of low frequency fluctuation (ALFF) or regional homogeneity analysis (ReHo) (Satterthwaite et al., 2012; Yang and Gohel, 2020).

Using task-based fMRI, Dietrich et al. (2001) and Stevens and Clark (2005) stressed the idea that the hemodynamic response function (HRF), whose canonical form lies behind most of the fMRI analysis, is modulated by blood estrogen and by estrogen therapy (Stevens and Clark, 2005). Plasma E_2_ could thus introduce confusion effects through probable modulation of the vascular compartment and of the neuro-vascular coupling, making it difficult to interpret fMRI results. Rangaprakash et al. (2018) showed that if the HRF variability, between individuals and cortical regions, is not considered in rs-fMRI, then up to 15% of error in functional connectivity estimation could occur and false connectivity detection could be increased. rs-fMRI is without a doubt a powerful tool that still presents some methodological challenges to implement in a robust manner.

## How Does Estrogen Treatment Influence the Default Mode Network Activity?

The DMN is characterized by the synchronous activation of brain regions as the medial prefrontal cortex, posterior cingulate cortex, precuneus, inferior parietal lobule, and inferolateral temporal cortex (Sood, 2013; Raichle, 2015) without external stimuli. It is presumably related to introspective and self-referential thought processes and becomes attenuated during goal-directed tasks (Ramírez-Barrantes et al., 2019), suggesting that its suppression during task execution favors the goal success (Fox et al., 2005; Hampson et al., 2006; Leech et al., 2011). The activation of DMN associates with processes like creativity and mind wandering, while abnormal activation and deactivation is related to psychiatric and medical conditions like anxiety, major depressive disorder, schizophrenia, and AD (Greicius et al., 2004; Anticevic et al., 2012; Whitfield-Gabrieli, 2012; Sunavsky, 2020).

Despite the important role of the RSN and the DMN in human cognition and wellbeing, little is known about physiological variability of the RSN connectivity across lifespan or between genders. A potential role of sex hormones on differential formation and activation of these RSNs would explain gender differences in cognitive tasks (Weis and Hodgetts, 2019; Pritschet et al., 2020), although the results are still inconsistent probably due to methodological differences between resting state studies. The function of DMN could be modulated across the menstrual cycle phases while it remains stable in men (Weis and Hodgetts, 2019). Predominantly composed by prefrontal areas that are very sensitive to sex hormones, DMN is presumably highly susceptible to menstrual cycle effects. In women, an increased DMN connectivity within the left middle frontal area in the menstrual phase as opposed to the follicular phase has been reported (Weis and Hodgetts, 2019). Other studies indicated that women in the luteal phase had reduced coherence between the left angular gyrus and the remaining network compared to women in the follicular phase. Oral contraceptive users in the active phase of their pill cycle showed reduced coherence between the left angular gyrus and the remaining network than women in follicular phase without contraceptive treatment (Petersen et al., 2014). Altered function of cell cycle or contraceptive methods could thus modify both abstract and self-referential reasoning and contribute to development of disease states involving DMN connectivity.

Estradiol determines central gender dimorphism (Ramírez-Barrantes and Marchant, 2016; Russell and Jones, 2019) and influences the performance in frontally mediated cognitive tasks, such as top-down cognitive control (Hjelmervik et al., 2014; Thimm et al., 2014). Hence, the withdrawal of E_2_ in the limbic system could be related with changes in mood, behavior, and cognition (Genazzani et al., 2007). In pre-menopausal women, during low E_2_ menstrual phase, activation of DMN in the left middle frontal area was increased (Weis and Hodgetts, 2019), while greater connectivity was demonstrated during the high-estrogen follicular menstrual phase (Petersen et al., 2014). These associations were observed with both studies having focused their investigation on the anterior frontoparietal network. Wang et al. showed an opposite effect of progesterone and estradiol in DMN (Wang et al., 2020). Estradiol, but not progesterone, could facilitate the medial prefrontal cortex-to-inferior parietal lobule functional connectivity, the posterior component of DMN (Wang et al., 2020). A huge study of the functional reorganization of brain networks during menstrual cycle found that progesterone is associated with negative connection in brain networks, while improved brain functional connectivity is mainly characterized by increased concentration of estradiol throughout the cycle (Pritschet et al., 2020). The activation of DMN probably contributes to memory problems described during the menopause transition. HT has been useful to improve verbal memory and activation of hippocampus in post-menopausal women compared to their counterparts who had never used HT (Ramírez-Barrantes et al., 2020).

Exogenous E_2_ has been associated with better cognitive performance during aging and reduced risk of AD where DMN is also involved (Henderson, 2006), but this potential effect is still in debate. Oral contraceptive pills have exhibited specific modulation of DMN in women with post-traumatic stress disorders (Wen et al., 2021). Ninety healthy women aged from 18 to 30 years underwent a 3-day fear conditioning and extinction paradigm. They were separated in two groups, namely, oral contraceptive regular users (*n* = 57) and never users of oral contraceptive pills (*n* = 33), and their DMN connectivity and attention networks were recorded by fMRI. E_2_ was beneficial to modulate attention and conscious processes promoting normal fear extinction learning and extinction memory retention. This kind of modulation by exogenous E_2_ has not been related to a unique network modulation, but to an activation of whole-brain functional connectivity, implying functionally distinct systems as the anterior component of DMN, i.e., regions with high levels of E_2_ receptors (Wen et al., 2021). Therefore, in addition to endogenous E_2_ across normal menstrual cycle, exogenous E_2_ could modulate DMN contributing processes like conscious awareness, affective learning, and memory consolidation in combination with attention network (Higgins et al., 2021).

## From Rapid to Long-Lasting Estrogen Effects

**Figure 1:**
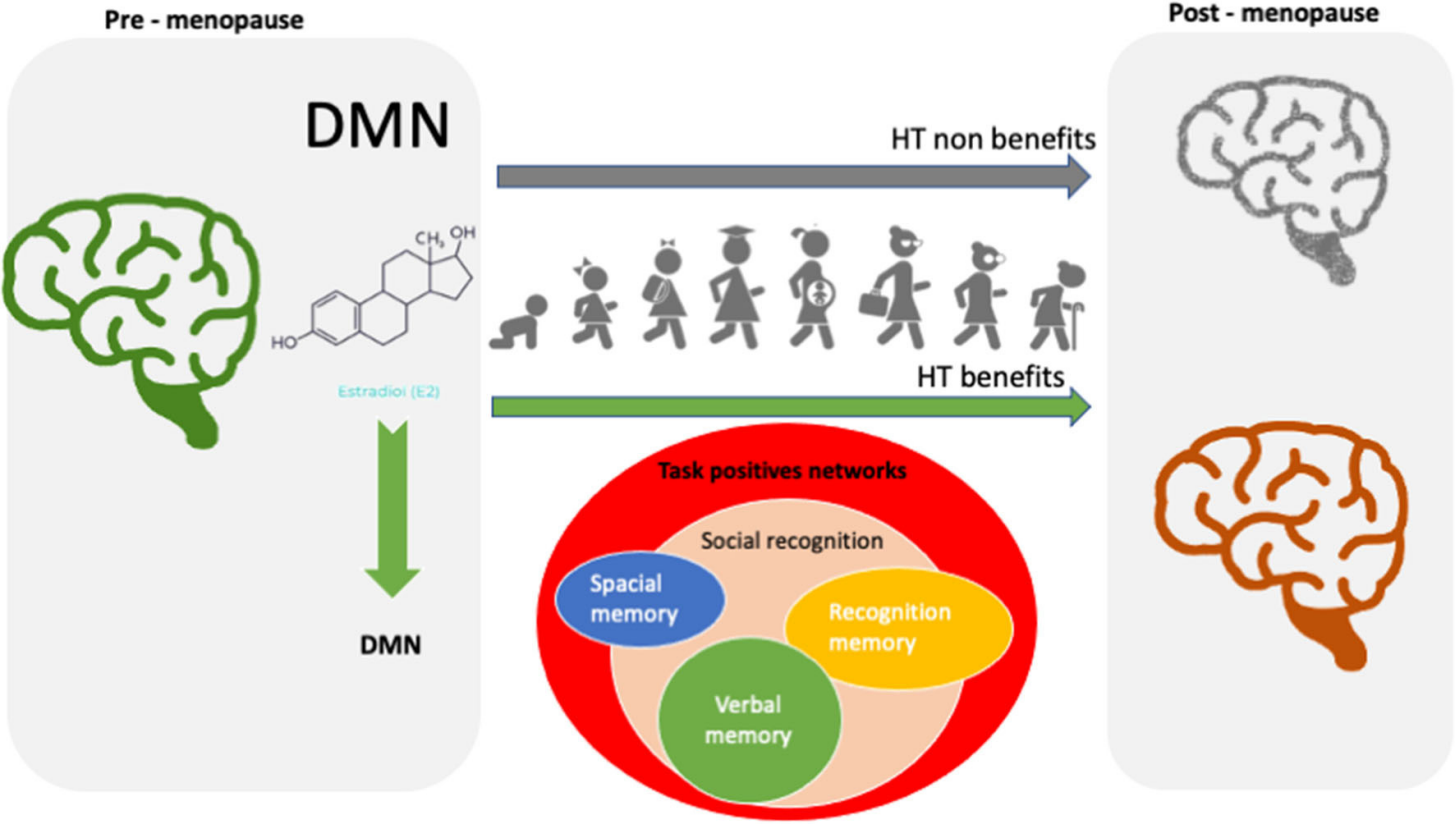
Rapid to long-lasting estrogen effects.

Different timescales underlie E_2_ effects (Figure 1). Genomic E_2_ mechanisms via ERs α and β trigger synthesis, release, and metabolism of neuropeptides and neuroesteroids. Non-genomic E_2_ actions trigger effects that appear in seconds to minutes (Sbarouni et al., 1997), modulate electrical excitability, and neuronal cell death (Zhao, 2007; Raz et al., 2008; Mukai et al., 2010; Ramírez-Barrantes et al., 2020). Rapid E_2_ mechanisms can modulate hippocampal memory consolidation within minutes of E_2_ exposure. In the context of direct acute E_2_ effect, we have demonstrated that it induces specific mitochondrial-mediated resistance to oxidative stress dependent of the function of membrane channel transient receptor potential cation channel subfamily V member 1 (TRPV1) (Ramírez-Barrantes et al., 2020). Altered mitochondrial proteostasis could thus impede the compensatory mechanisms against cell damage. The rapid modification of the activity of non-classical E_2_ receptors, such as TRPV1, might be critical for the maintenance of intracerebral functional connectivity during acute E_2_ stimulation. Future clinical trials focused on these rapid signal changes following estrogen exposure may identify predictors of long-lasting HT effectiveness.

## Author Contributions

IM and PO conceived the idea and wrote the manuscript. SC, JM-P, RS-Z, and RR-B developed contents in rs-fMRI, animal models, connection with human studies, and DMN, respectively. LA reviewed critically neurological topics. CC developed figures. All authors reviewed critically the final version for intellectual contents.

## Conflict of Interest

The authors declare that the research was conducted in the absence of any commercial or financial relationships that could be construed as a potential conflict of interest.

## Publisher's Note

All claims expressed in this article are solely those of the authors and do not necessarily represent those of their affiliated organizations, or those of the publisher, the editors and the reviewers. Any product that may be evaluated in this article, or claim that may be made by its manufacturer, is not guaranteed or endorsed by the publisher.
